# Multimodality Treatment in Metastatic Gastric Cancer: From Past to Next Future

**DOI:** 10.3390/cancers12092598

**Published:** 2020-09-11

**Authors:** Alessandro Parisi, Giampiero Porzio, Corrado Ficorella

**Affiliations:** 1 Medical Oncology, St. Salvatore Hospital, University of L’Aquila, 67100 L’Aquila, Italy; porzio.giampiero@gmail.com (G.P.); corrado.ficorella@univaq.it (C.F.); 2 Department of Biotechnology and Applied Clinical Sciences, University of L’Aquila, 67100 L’Aquila, Italy

**Keywords:** advanced gastric cancer, chemotherapy, targeted therapy, immunotherapy, surgical treatment, supportive care, locoregional treatment

## Abstract

**Simple Summary:**

Gastric cancer remains an incurable disease in most of the cases. Anyway, the progress achieved over the last decade in terms of knowledge of its biology and available therapeutic options, together with a greater attention to the concept of supportive care, led to a progressive and incremental survival benefit in metastatic gastric cancer patients. In this review we summarize the current standard management and the major completed or ongoing clinical trials involving systemic, surgical or locoregional treatment of metastatic gastric cancer along with emerging concepts likely to improve patients’ outcome in the next future.

**Abstract:**

Gastric cancer (GC) still remains an incurable disease in almost two-thirds of the cases. However, a deeper knowledge of its biology in the last few years has revealed potential biomarkers suitable for tailored treatment with targeted agents. This aspect, together with the improvement in early supportive care and a wiser use of the available cytotoxic drugs across multiple lines of treatment, has resulted in incremental and progressive survival benefits. Furthermore, slowly but surely, targeted therapies and immune checkpoint inhibitors are revising the therapeutic scenario even in metastatic GC and especially in particular subgroups. Moreover, important study results regarding the possible role of an integrated approach combining systemic, surgical, and locoregional treatment in carefully selected oligometastatic GC patients are awaited. This review summarizes the state-of-the-art and the major ongoing trials involving a multimodal treatment of metastatic GC.

## 1. Introduction

Gastric cancer (GC) is the fifth malignancy and the third cause of cancer death worldwide, according to the global cancer statistics presented in 2018 (GLOBOCAN 2018 [1]). Despite the improvements in the perioperative treatment, about 50% of resected patients with curative intent eventually relapse, while 80% of patients present a de novo unresectable or metastatic disease [2]. In this setting, fluoropyrimidine- and platinum-based systemic treatment represents the standard of care, with median overall survival (mOS) of about 10 months in human epidermal receptor 2 (HER-2) negative disease [3], extending to about 15 months in HER-2 positive disease with the addition of the monoclonal antibody (mAb) trastuzumab [4]. After almost a decade of plateau in survival in metastatic GC (mGC), the advent of vascular endothelial growth factor receptor-2 (VEGFR-2) inhibitors such as ramucirumab [5,6] and apatinib [7], as well as the innovative oral cytotoxic trifluridine-tipiracil [8] and, especially in particular subgroups of patients (i.e., programmed death [PD] ligand 1 [PD-L1] positive, microsatellite instability [MSI]-high, Epstein–Barr virus [EBV], positive or high tumor mutational burden [TMB]), immune checkpoint inhibitors (ICIs) [9,10] led to progressive incremental survival advantages.

On the other hand, if surgery represents the cornerstone in the curative setting, its role in the metastatic disease is associated with controversial results [11,12,13,14] as well as the impact of other locoregional strategies [15,16,17,18,19]. In this regard, the concept of “oligometastatic” GC, stating a disease characterized by limited tumor burden (i.e., M1 with retroperitoneal lymph nodes and/or one potentially resectable incurable site), is taking place as emerging clinical entity, distinct from extensively mGC (M1 patients other than oligometastatic) in terms of both treatment plan (multimodal treatment vs. systemic treatment alone, respectively) and survival (mOS of about 31 months vs. 9–11 months, respectively) [12,13,14,15,20].

Furthermore, a growing amount of evidence highlighted the importance of the best supportive care (BSC), especially in such cancer patients at high risk of malnutrition, loss of body composition parameters, and sarcopenia, with detrimental effects on safety and outcome of both systemic and surgical treatments [21,22,23].

In this article, we review the major advances in systemic, surgical, and locoregional treatment of mGC in parallel with the evolution of the role of BSC in this disease.

## 2. Systemic Treatment

### 2.1. Standard of Care

#### 2.1.1. First-Line Treatment

The issue regarding type and intensity of the first-line treatment in mGC has long been debated and investigated. A combination chemotherapy demonstrated to improve survival and quality of life (QoL) compared to single-agent fluoropyrimidine [3].

In HER-2 negative patients, platinum/fluoropyrimidine doublet chemotherapy is the preferred regimen both in Western and Eastern countries, reaching a mOS of about 10 months, with cisplatin and 5-fluorouracil (5-FU) replaceable by oxaliplatin and capecitabine, respectively, according to their non-inferiority and better safety profile [24]. Irinotecan/5-FU combinations represent a further valuable first-line alternative, resulting in at least non-inferior as efficacy and with a better safety profile if compared to a platinum/fluoropyrimidine regimen with or without epirubicin [25].

In HER-2 positive disease (15–20% of the cases), according to the results of the randomized phase III TOGA trial, adding the anti-HER2 monoclonal antibody trastuzumab to a fluoropyrimidine/cisplatin doublet has shown to improve the overall response rate (ORR) (47% vs. 35%, *p* = 0.0017), progression-free survival (PFS) (6.7 vs. 5.5 months, HR = 0.71, *p* = 0.0002), and OS (13.8 vs. 11.1 months, HR = 0.74, *p* = 0.0046) [4], with the greatest benefit in OS in favor of the strong (3+) HER-2 overexpressing tumors (16 vs. 11.8 months, HR = 0.65, *p* = 0.0046).

The role of adding a third cytotoxic agent to a doublet with platinum/fluoropyrimidine as first-line treatment of mGC has been widely investigated and if the advantage of epirubicin is still controversial [26], several docetaxel-based triplet regimens have been developed. In general, with respect to a doublet, a triplet regimen allows higher ORR (RR: 1.25, 95%CI 1.09–1.44) at the price of major incidence of adverse events (AEs), particularly severe mucositis (9.7% vs. 4.7%), thrombocytopenia (6.2% vs. 3.8%), and infection (10.2% vs. 6.4%), leading to a statistically significant but clinically very low relevant gain in both PFS (HR = 0.8, 95%CI 0.69–0.93) and OS (HR = 0.90, 95%CI 0.83–0.97) [27]. Variants of the standard DCF regimen, combination of docetaxel, cisplatin, and fluorouracil first tested in the V325 phase III trial [28], as dose-modified DCF (mDCF) [29] or even non cisplatin-containing regimens such as FLOT [20], TEF [30], or similar [31] (combinations of docetaxel, oxaliplatin, and fluoropyrimidine) showed a more favorable toxicity profile. A Western phase III study is currently comparing TFOX (similar to the TEF regimen) with FOLFOX as first-line treatment in mGC (GASTFOX: NCT03006432), while an Eastern phase III study is investigating in the same setting, the role of a triplet combination of irinotecan, fluorouracil, and oxaliplatin [NCT04358354], as already tested in metastatic colorectal cancer (mCRC) [32,33].

To date, it is crucial to select the patient fit for an intensive regimen and features of both patient (age, comorbidities, expected QoL) and disease (tumor burden, symptoms) play a central role in the decision-making process [31]. Therefore, a triplet chemotherapy, can be justified in those patients less likely to receive second-line treatment, with high tumor burden and symptomatic disease needing rapid tumor shrinkage, with careful management of the toxicity profile. On the other hand, a sequential strategy should be preferred in those patients with low tumor burden and asymptomatic disease, in favor of a better QoL profile and a reduced risk of cross-resistance in view of a potential taxane-based second-line treatment.

#### 2.1.2. Second- and Further Line Treatment

Indeed, for mGC patients progressed to a first-line treatment and maintaining acceptable performance status, the human monoclonal antibody (mAb) anti-VEGFR2 ramucirumab has been shown to improve OS both alone if compared to BSC (5.2 vs. 3.8 months, HR = 0.77, *p* = 0.047) and combined with paclitaxel with respect to paclitaxel alone (9.6 vs. 7.4 months, HR = 0.80, *p* = 0.017), as second-line treatment as the result of two international randomized double-blind phase III trials (REGARD and RAINBOW, respectively) [5,6]. Both efficacy and safety data of ramucirumab have been confirmed in “real-life” settings [34,35].

Besides ramucirumab, single-agent taxane (paclitaxel and docetaxel) and irinotecan showed increased survival compared to BSC as second-line treatment in mGC with a median survival gain ranging from 1.4 to 2.7 months among individual studies and with different safety profiles [36,37,38].

Notable, a doublet chemotherapy taxane/irinotecan plus platinum and fluoropyrimidine provides no gain in survival if compared to taxane/irinotecan monotherapy as second-line treatment in mGC and is associated with increased toxicity [39].

Regarding third-line treatment in mGC, the first prospective evidence supporting the role of a systemic therapy in this setting came from a randomized phase III trial comparing the VEGFR-2 tyrosine kinase inhibitor (TKI) apatinib over placebo, (mOS 6.5 vs. 4.8 months, respectively, HR = 0.70, *p* = 0.149) in Eastern mGC patients pretreated with two or more lines of chemotherapy [7]. Regrettably, these results were not confirmed in the same clinical setting in Eastern populations in the phase III ANGEL trial [40].

Then, another two randomized phase III trials showed a statistically significant improvement in OS and QoL for the novel oral cytotoxic trifluridine/tipiracil over placebo (5.7 vs. 3.6 months, HR = 0.69, *p* = 0.0005) in a global population [8] and for the anti-PD1 agent nivolumab over placebo in Eastern patients [9]. Furthermore, according to the results of the phase II KEYNOTE 059 study [41], the Food and Drug Administration (FDA) in the USA approved pembrolizumab in mGC patients with combined positive score (CPS) for PD-L1 ≥ 1% and/or MSI-high tumors.

### 2.2. Targeted Agents

GC frequently harbors genetic aberrations and genomic instability as amplifications and co-amplifications of genes encoding for receptor tyrosine kinases (RTKs) (RAS, HER-2, and MET), for cell cycle mediators (such as proteins related to DNA damage repair) or even interspersed among pathways related to angiogenesis, all molecular hallmarks of GC involved in tumor initiation, progression, and treatment resistance [2].

However, with the exception of trastuzumab and ramucirumab in the first- and second-line setting respectively, drug development in targeted agents provided disappointing results in mGC phase III clinical trials targeting *HER-2* with TKI [42,43], antibody-drug conjugate (ADC) [44], or dual-blockade [45], *EGFR* [46,47], *MET* [48,49], *PI3K/mTOR* [50,51], *STAT3* [52], *MMP9* [53], and *PARP* [54] (Table 1).

This failure is assumed to be caused by the highly heterogeneous both intra- and interpatient histologic features and molecular biology of GC as recently highlighted by The Cancer Genome Atlas (TCGA) classification [55], and therefore by the lack of a proper biomarker selection. One solution to overcome the obstacle of the intratumor heterogeneity may be the use of circulating tumor DNA (ctDNA) to detect aberrations of genomic instability not detecting by tumor sampling and analysis [56,57]. Indeed, for example, a small subset of mGC seems to be mainly driven by the oncogenic EGFR pathway and anti-EGFR treatment with cetuximab may result in a relevant clinical benefit in heavily pretreated patients [58].

Intriguingly, in contrast with the positive results reached in the second-line, ramucirumab did not improve OS as first-line treatment [58] and the monoclonal antibody targeting VEGF-A bevacizumab failed to improve OS in the same setting [50,51,52,53,54,55,56,57,58,59,60]. These discrepancies between colorectal cancer (CRC), in which angiogenic inhibition is a SOC treatment both in the first- and second-line setting [61], and GC may be related to deep differences in biology, including tumor microenvironment and molecular pathways of resistance to VEGF inhibition (stromal signaling pathways related to FGF, PIGF, and PDGF and oncogenic signaling pathways related to RAF, RET, and cKIT) [2,55,56].

On the other hand, the VEGFR-2 TKI apatinib improved OS as third-line treatment in the Eastern population [7] but failed in the same setting in Western patients [40] (Table 2). These results may be at least in part explainable with differences in both prognostic factors and biological profiles between the two populations [2,55].

Ongoing phase III trials with the VEGFR1-2-3 TKI fruquintinib (FRUTIGA: NCT02773524) and the multi-TKI regorafenib (INTEGRATE II: NCT02773524) are assessing the value of a deeper both angiogenic, stromal, and oncogenic inhibition in mGC, but to date, further studies are warranted to identify potential molecular biomarkers for selecting mGC patients who should benefit most from the addition of VEGF inhibitors.

Regarding HER-2 targeted therapy, with the exception of the significant OS benefit provided by the addition of trastuzumab to chemotherapy in the first-line setting [4], the HER-1 TKI lapatinib, the HER-2 dual-blockade with pertuzumab and trastuzumab or the ADC trastuzumab emtansine (T-DM1) led to negative results in different line settings and combinations in HER-2 positive mGC [41,42,43,44] (Table 1).

Notably, in the randomized phase II DESTINY-Gastric01 trial, presented at the 2020 ASCO Virtual Meeting, the ADC trastuzumab deruxtecan (DS-8201a) provided a significant improvement in both ORR (51% vs. 14%, *p* < 0.001) and OS (12.5 vs. 8.4 months, *p* = 0.01) compared to physician’s choice chemotherapy in heavily pretreated HER-2 positive Eastern mGC patients, with myelosuppression and interstitial lung disease as notable toxic effects [62]. Data from the Western population (DESTINY-Gastric02: NCT04014075) and confirming phase III trials are awaited.

Among other interesting targeted agents, as proof of the importance of a proper molecular selection, the tight junction protein claudin 18.2 (CLDN18.2) inhibitor zolbetuximab showed improved PFS and OS in association with chemotherapy as first-line treatment of CLDN18.2-positive mGC in a phase II trial [63]. Two phase III confirmatory trials are currently ongoing (SPOTLIGHT: NCT03504397 and GLOW: NCT035653507) (Table 1).

### 2.3. Immunotherapy

Breakthrough results from immune checkpoint inhibitors (ICI) have recently opened the doors to a new era of cancer immunotherapy, leading to a paradigm shift in cancer treatment [64]. Particularly, GC seems to be an “immunologically hot” tumor, associated with overexpression of immune checkpoint proteins (such as PD-L1, in up to 65% of the GCs), high TMB (i.e., the total number of mutations per coding area of a tumor genome), and immune system evasion, providing the rationale for immunotherapy with PD-1/PD-L1 inhibitors, alone or combined with chemotherapy or targeted agents [65].

In the landmark phase III ATTRACTION-02 trial [9], the human mAb anti-PD1 nivolumab significantly prolonged OS over placebo (5.2 vs. 4.1 months, HR = 0.63, *p* < 0.0001) in Asian mGC patients progressed to almost two previous regimens, reaching a 3-year OS of 5.6% and 1.9%, respectively, suggesting that a proportion of mGC patients achieved durable OS benefit from nivolumab. The toxicity profile was manageable with mainly mild to moderate AEs including diarrhea, fatigue, pruritus, and rash. Of note, a longer OS was observed in those patients who experienced treatment-related AEs of special interest (endocrine, gastrointestinal, hepatic, hypersensitivity reaction, pulmonary, renal, or skin) compared with those who did not (2-year OS of 20% and 0%, respectively) [66]. This is in line with previous reports in other cancer settings [67]. Intriguingly, no difference was seen according to PD-L1 status (measured with the tumor proportion score [TPS]) even if PD-L1 immunohistochemistry threshold for positivity was set at 1% and tumor samples were available in only about 40% of patients.

Similar data were obtained in Western populations even if phase III data are lacking. The phase I-II CheckMate-032 trial tested nivolumab alone or combined with the fully human mAb inhibitor of cytotoxic T-lymphocyte associated protein-4 (CTLA-4) ipilimumab in 160 heavily pretreated mGC patients, reaching ORR of 12% and 24% and G3–4 AEs of 17 and 47%, respectively [68]. Intriguingly, these activity results were obtained regardless of PD-L1 status. The ongoing phase III CheckMate-649 trial (NCT02872116) is testing this association as fist-line treatment of HER2-negative mGC (Table 2).

On the other hand, the human mAb anti-PD1 pembrolizumab was first globally tested in the large phase II KEYNOTE-059 trial, reaching higher ORR (15.5% vs. 6.4%) and longer duration of response (DOR, 16.3 vs. 6.9 months) in PD-L1-positive (defined as combined positive score [CPS] ≥ 1%) than in PD-L1-negative mGC as third- or further-line treatment [41]. Even then, phase III study data are awaited (Table 2).

At least in part in line with these data, encouraging results were found in phase III trials evaluating the efficacy of monotherapy with PD-1/PD-L1 inhibitors compared with chemotherapy. In a recent 2-year update analysis of the KEYNOTE-061 trial, pembrolizumab monotherapy provided a trend toward improved OS as second-line treatment in mostly Western PD-L1-positive (CPS ≥ 1%) mGC compared to paclitaxel (mOS 9.1 vs. 8.3 months, HR = 0.81, *p* = 0.03) [69]. An even more pronounced benefit in terms of OS, ORR, and DOR from pembrolizumab over paclitaxel was seen in certain subgroups (performance status 0, CPS ≥ 10% and MSI-high). On the other hand, in the global JAVELIN Gastric 300 trial, the PD-L1 inhibitor avelumab failed to improve survival over physician’s choice chemotherapy (mOS: 4.6 vs. 5.0 months, *p* = 0.81, respectively) as third-line treatment in 371 mGC patients, regardless of PD-L1 status (TPS ≥ 1%) [70]. Furthermore, the phase III JAVELIN Gastric 100 (NCT02625610), evaluating the role of avelumab as first-line maintenance treatment after an induction phase with XELOX/FOLFOX provided negative results in PD-L1 positive (TPS ≥ 1%) mGC patients [71].

Combining immunotherapy and chemotherapy might be of benefit in improving immunogenicity, especially increasing TMB with platinum agents [72]. In the global phase III KEYNOTE-062 trial [73], 763 HER-2 negative and PD-L1 positive (CPS ≥ 1%) untreated mGC patients were randomized to three arms with two comparisons: pembrolizumab alone vs. cisplatin and fluoropyrimidine (PF) (non-inferiority) and pembrolizumab plus PF vs. PF (superiority). Non-inferiority of pembrolizumab was demonstrated in terms of OS in CPS ≥ 1% (mOS 10.6 vs. 11.1 months, HR: 0.91, non-inferiority margin: 1.2), especially in CPS ≥ 10% (mOS 17.4 vs. 10.8 months, HR: 0.69), even if ORR and PFS were worse in the pembrolizumab arm in CPS ≥ 1% but better in CPS ≥ 10% patients. On the other hand, chemotherapy plus pembrolizumab was not formally superior to chemotherapy alone in terms of OS even if a favorable trend was seen both in CPS ≥ 1% (mOS 12.5 vs. 11.1 months, HR: 0.85, *p* = 0.046) and CPS ≥ 10% (mOS 12.3 vs. 10.8, HR: 0.85, *p* = 0.158) patients. In an exploratory analyses of this trial conducted among 50 MSI-high patients, median OS was not reached in both pembrolizumab arms compared with 8.5 months with chemotherapy alone, while ORR was almost doubled in both pembrolizumab arms compared to chemotherapy alone [74]. These data further support the FDA approval of pembrolizumab for PD-L1 ≥ 1% as well as agnostic indication for unresectable or metastatic mismatch repair deficient (dMMR) and/or MSI-high solid tumors, including GC, with no alternative options. On the other hand, the amount of negative results stresses the need to better define potential predictive biomarkers for immunotherapy in clinical trials with ICI (e.g., CPS maybe more reliable than TPS as assessor of PD-L1 status; PD-L1 threshold of 10% maybe more realistic than 1%). For this purpose, it might be useful to refer to the four genomic subtypes of GC as defined by TGCA (EBV-positive [8%], characterized by a higher prevalence of DNA hypermetilation, *PD-L1/L2* amplification, *PIK3CA* and *ARID1A* mutations; MSI-positive [22%], exhibiting high TMB, *MLH1* promoter hypermetilation and *PIK3CA* mutations; genomically stable [GS, 20%], harboring *CDH1* and *RHOA* mutations and CLDN18-ARHGAP rearrangements; chromosomal instability positive [CIN, 50%], harboring TP53 mutations as well RAS receptor tyrosine kinase pathway (i.e., VEGFA, EGFR, HER2-3, FGFR2) and cell cycle mediators (i.e., CDK6) amplifications) [55]. As told previously, MSI-high GC, maybe in relation with the dense concentration of CD8+ tumor-infiltrating lymphocytes (TILs) stimulated by immunogenic neoantigens in turn generated by a hypermutational load (TMB), are characterized by higher ORR compared with non-MSI-high GC [41,74,75]. The EBV subtype, characterized by increased PD-L1 expression in both tumor cells and TILs, seems to be even more responsive to ICI [75]. Gut microbiome has been shown to be associated with efficacy of anti-PD1 mAb in different types of cancer [76] and the DELIVER trial (JACCRO GC-08, UMIN000030850) is investigating the role of novel immune-related biomarkers (gut microbiome, genetic polymorphism, gene expression, and metabolome in plasma) in mGC patients treated with nivolumab.

Several trials are further investigating the role of PD-1/PD-L1 inhibitors in combination with chemotherapy as first- or second-line treatment for mGC as well as the potential role of combination with targeted agents (Table 2). Indeed, it is well recognized that tumor neovascularization promoted by tumor-induced angiogenic factors as VEGF can activate immunosuppressive cells as regulatory T cells (Treg) and tumor-associated macrophages (TAM), in turn involved in tumor progression, invasion, and angiogenesis downregulating anti-tumor TILs, especially CD8+ Cytotoxic T-Lymphocytes (CTLs) [77]. Antiangiogenic agents may restore the immune antitumor activity disrupting the VEGF/VEGFR axis inhibition in the tumor microenvironment [78]. Promising activity and efficacy results derived from phase I-II clinical trials combining ramucirumab with nivolumab (NivoRam) [79] or pembrolizumab (KEYNOTE-098) [80], or nivolumab with regorafenib (REGONIVO) [81], or lenvatinib plus pembrolizumab [82]. Exciting the combinations with chemotherapy evaluating the association of paclitaxel, ramucirumab and avelumab (RAP: NCT03966118) or pembrolizumab (SEQUEL: NCT04069273).

Ultimately, trastuzumab seems to upregulate the expression of PD-L1 and enhance gene expression signature of immune infiltration [83], providing a rationale for combination with ICI. In the phase II 16-937 trial [84], 37 HER2-positive mGC treated with XELOX and trastuzumab in combination with pembrolizumab as first-line therapy, reaching an ORR and a 6-month PFS of 91% and 70%, respectively. The confirmatory phase III KEYNOTE-811 (NCT03615326) trial is ongoing (Table 2).

## 3. Surgery and Locoregional Treatments

If surgery represents the cornerstone of treatment in the early or locally advanced GC, eventually associated with neoadjuvant [85] or adjuvant [86] systemic treatment according to local guidelines and clinical practice, its role in the metastatic disease is still far from clear.

Theoretically, gastrectomy might reduce a large and immunosuppressive tumor burden, removing the source of new metastases and improving symptoms related to the primary tumor such as bleeding, perforation, and obstruction. On the contrary, gastrectomy could lead to disease progression inducing immunosuppression, delaying systemic treatment delivery as a consequence of postoperative complications, or making systemic treatment less tolerable.

The role of the surgical resection of the primary tumor has been investigated in the Asian phase III REGATTA trial [11], in which primary tumor resection followed by systemic treatment provided no survival benefit compared to systemic treatment alone (mOS 14.3 vs. 16.6 months, respectively, HR = 1.09, *p* = 0.70) in mGC patients with a single non-curable factor, resulting even detrimental. It is important to underline that in this trial, resection of metastatic lesions was not allowed and this could at least in part have influenced the negative results.

Indeed, a growing amount of evidences showed a possible role of both gastrectomy and metastases treatment, especially in case of liver involvement, occurring in about 40% of the cases of synchronous disease, 70% of which confined to the liver with no diffusion to other organs [87]. In a recent systematic review and pooled analysis of 39 both Eastern (30) and Western (9) studies published over 25 years and including 991 mGC patients who underwent resection for liver metastases, the median 1-year, 3-year, and 5-year survival in Eastern/Western populations was 73/59%, 34/24.5%, and 27/16.5%, respectively [14]. Liver metastases resection was associated with a significantly improved overall survival (HR = 0.5; *p* < 0.001) and a median 30-day morbidity and mortality of 24% and 0%, respectively [14]. Even if deriving from small and retrospective studies, heterogeneous for selection criteria and for the eventually performed pre- or postoperative systemic treatment, these data support the possibility for carefully selected “oligometastatic” GC patients (i.e., patients with solitary and unilobar liver-only metastasis, R0 resectable, with complete removal of primary gastric tumor and lymph nodes for synchronous metastases) to achieve long-term benefit from a multimodality treatment strategy. In the arm B of the phase II AIO/FLOT3 trial [20], a three-arm, prospective, non-randomized study, 60 patients with limited mGC (i.e., retroperitoneal lymph node metastases with no diffuse nor symptomatic nor clinically detectable peritoneal carcinomatosis, with or without one of the following: <5 liver lesions or Krukemberg tumors or adrenal gland metastases) received 4 cycles of FLOT regimen (5-fluorouracil, oxaliplatin, and docetaxel) followed by radical macroscopic surgery of both primary tumor and metastases, whenever feasible, and then 4 subsequent postoperative FLOT cycles (arm B). Arm A and arm C of the study consisted of patients with locally advanced and widely metastatic disease, respectively, and received perioperative (arm A) and palliative (arm C) FLOT. In the arm B, 36 (60%) patients underwent surgery, with improved survival compared to those who did not (mOS 31.3 vs. 15.9 months, respectively) or those with extensive metastatic disease (mOS 10.7 months). The ongoing phase III RENAISSANCE/AIO-FLOT5 trial (NCT02578368) will confirm the role of a multimodal treatment over systemic treatment alone in oligometastatic GC patients (Table 3).

Besides resection, other locoregional treatments such as radiofrequency ablation (RFA), microwave ablation (MWA) [16], hepatic artery infusion chemotherapy (HAIC) [17], transarterial chemoembolization (TACE) [18], and stereotactic body radiotherapy (SBRT) [19] have been investigated in mGC. Overall, these treatments were less invasive and associated with less minor and major complications, resulting at least non-inferior if compared to resection in highly-selected mGC patients with small (e.g., <3–5 cm in size for RFA and MWA) liver-limited lesions [16]. Of course, proper designed studies are warranted to better define the role of these treatments as potentially alternative or complementary to surgery and systemic treatment in selected mGC patients.

Peritoneal metastases occur in above 40% of synchronous disease and up to 46% of metachronous cases, in above 70% of the patients with no other organs involvement [88]. Malignant ascites, bowel obstruction, and nutritional impairment caused by peritoneal carcinomatosis lead to both poor survival and QoL in mGC patients [89]. To improve both survival and symptoms control for GC patients with peritoneal metastases, cytoreductive surgery (CRS) followed by hypertermic intraperitoneal chemotherapy (HIPEC) have been investigated during the last decades [88]. CRS is a complex surgical procedure including peritonectomy and resection of involved viscera with the aim of removing macroscopic disease [90]. The biological rationale for intraperitoneal delivery is based on preclinical evidences of a pharmacokinetic advantage explained by the existence of a peritoneal-plasma barrier, allowing a high concentration gradient of chemotherapeutic drugs between the peritoneal cavity and the systemic circulation [91]. An additional advantage to intraperitoneal chemotherapy administration is that blood drainage from the peritoneal cavity is through the portal system, providing a detoxifying “first-pass” effect, reducing systemic toxicity [92]. Furthermore, experimental and clinical evidences highlight how malignant cells are selectively destroyed by hyperthermia in the range of 41–43 °C [93], especially when selected drugs (i.e., taxanes, platinum compounds, mitomycin C, and anthracyclines) are used [94]. In a recent meta-analysis of 11 randomized and 21 non-randomized comparative studies published over 30 years and including 2520 mGC patients, CRS plus HIPEC is associated with longer mOS compared to the control group (11.1 vs. 7.0 months) in selected patients with positive peritoneal citology only, or limited nodal involvement, or with extensive carcinomatosis in which a radical cytoreductive surgery can be achieved. This benefit is associated to significantly higher risk of postoperative complications (RR: 2.15), in particular, respiratory and renal failure [15]. Because of the technical complexity, the high risk of postoperative complications and since the likelihood of a complete macroscopic cytoreduction is related to a surgeon’s experience, CRS and HIPEC should only be performed in specialized high-volume centers [93]. Recently, the most significant development in intraperitoneal-directed therapy is pressurized intraperitoneal aerosol chemotherapy (PIPAC), a minimally invasive procedure, generally safe and well tolerated, capable of achieving a more uniform distribution and deeper peritoneal penetration in gaseous state when compared to liquid chemotherapy [94]. Data on efficacy and safety of PIPAC in GC patients with peritoneal metastases remains limited, with only 4 studies (2 retrospective studies and 2 phase II trials), comprising a total of 274 PIPAC procedures administered in 119 GC patients [94,95,96,97]. In these studies, mOS rates, major complication rates, and mortality rates ranged between 4.0–19.5 months, 0–29% and 0–8.3%, respectively [94,95,96,97]. The multicenter, international online documentation of indications and results of PIPAC (PIPACRegis—NCT03210298), is an international prospective patient registry intended to collect clinical data from 1000 cancer patients undergoing PIPEC.

Results of ongoing randomized phase II-III clinical trials evaluating the efficacy of multimodal approaches in mGC patients are highly awaited (Table 3).

## 4. Sarcopenia, Cachexia, Malnutrition, and Supportive Care

GC is among the leading oncologic causes of sarcopenia, a complex syndrome characterized by progressive and generalized loss of skeletal muscle mass and strength, fatigue, and metabolic disorders, ultimately resulting in a condition of body weight loss and then cachexia in 85% of the cases [98,99]. In turn, cachexia, defined as a body weight loss more than 5% within 12 months or less, contributes substantially to morbidity and mortality in cancer patients, accounting for more than 20% of cancer deaths [98,100]. The cachectic state is a life-threatening syndrome encompassing skeletal muscle and adipose tissue loss, and it is frequently associated with muscle atrophy and a deregulated metabolic state characterized by insulin resistance, reduced anabolic activity, elevated cortisol levels, increased basal energy expenditure, and resistance to conventional nutritional support [101]. It results from an extensive interaction between proinflammatory cytokines (i.e., IL-1, IL-6, TNF-a, and others) and neuroendocrine factors generated by both tumor and host cells [100]. Additionally, cachexia-associated cytokines are able to cross the blood-brain barrier and modify the activity of hunger regulatory systems. As a result, from 15% to 40% of cancer patients with cachexia often develop anorexia [101], establishing a vicious cycle of malnutrition, sarcopenia, and cachexia.

Sarcopenia is a well-known independent predictor of short- and long-term postsurgical outcome in GC [21,102], associated to higher postsurgical complication rate (i.e., infection rate), longer hospitalization, more frequent need of mechanical ventilation, and a greater number of hospital readmissions, ultimately leading to poor DFS and OS. Likewise, sarcopenia is associated with toxicity in GC patients undergoing perioperative systemic treatment for early stage of disease [22,23,103] or multiple lines of therapy in the metastatic setting [35], leading to early discontinuation of treatment, reduced efficacy of antineoplastic agents, and poor prognosis.

With these thought in mind, it is important to carry out an overall assessment of the nutritional status and symptoms burden of the mGC patient in each moment of his natural history, using well-recognized clinical (i.e., Mini Nutritional Assessment (MNA) [104] and PERSONS score [105,106] in addition to body mass index (BMI) and body surface area (BSA) [98]), biochemical (i.e., C-reactive protein and albumin ratio [107]) and instrumental (i.e., radiological evaluation of skeletal muscle mass, skeletal muscle index, and skeletal muscle radiodensity [108,109]) parameters, to promptly identify, grading, monitoring, and treating risk and causes of malnutrition, sarcopenia, and cachexia. Indeed, the early integration of nutritional screening, personalized nutritional support, and prehabilitation programs with physical exercise, has been shown to increase muscle mass and prevent or limit sarcopenia, with better short- and long-term post-gastrectomy outcomes [110]. In mGC patients frequently affected by impaired gastrointestinal function and inadequate food intake as a consequence of dysphagia or obstruction, nutritional support provided by oral, enteral, and/or parenteral nutrition may improve patients’ QoL and adherence to systemic therapies [111]. Many molecules, including anabolic agents and anti-inflammatory drugs, have been developed to limit sarcopenia and cachexia. Megestrol acetate and medroxyprogesterone acetate, alone [112] or combined with other compounds such as formeterol acetate and mirtazapine [113] demonstrated to stimulate appetite and weight gain, downregulating proinflammatory citokines. Similarly, corticosteroids could improve appetite, asthenia, energy, and wellness [114]. If the ongoing phase III EPIC-1511 trial (NCT2853474) will better define the importance of the early integration of supportive care in addition to SOC treatment with respect to SOC alone and palliative care as needed in upper gastrointestinal cancer patients, there is no doubt that new drugs to counter body mass loss and increase the efficacy of nutritional support in GC patients are urgently required.

## 5. Concluding Remarks

In recent years, multiple targeted agents and immunotherapy drugs have been investigated in mGC, providing mostly negative results in unselected or poorly selected patients, but with evidence of promising survival and clinical benefit for certain subgroups. On the other hand, “oligometastatic” GC would increasingly seem to be a clinical entity with distinct prognosis and therapeutic implications, able to benefit from a multimodality approach including systemic, surgical, and locoregional treatments. Therefore, it is imperative to find and define specific and proper clinical biomarkers for targeted and immunotherapy agents and selection criteria for surgical and locoregional therapies. At the same time, early evaluations of nutritional status and timely nutritional support are key aspects capable of improving prognosis in all the phases and settings of GC disease.

## Figures and Tables

**Table 1:** List of completed/ongoing phase III or II/III trials with targeted agents in metastatic gastric cancer.

Study name [reference]	Year	Country	Ph	Line	N	Target	Drug	Selected Population	Study Intervention exp/cont	OS-PFS (months) exp/cont	Grade 3–4 AEs exp (%)	Res
ToGA [4]	2010	Inter	III	1°	594	HER2	Trastuzumab	HER2 pos (IHC3+, FISH+)	FP (XP) + trastuzumab FP (XP)	13.8—6.7 11.1—5.5	nausea (67) vomiting (50) neutropenia (53)	Pos
TRIO-013/LOGiC [42]	2016	Inter	III	1°	545	HER1-2	Lapatinib	HER2 pos (FISH+)	XELOX + lapatinib XELOX + PBO	12.2—6.0 10.5—5.4	diarrhea (12) nausea (6) vomiting (6)	Neg
TyTAN [43]	2014	Asia	III	2°	261	HER1-2	Lapatinib	HER2 pos (FISH+)	PTX + lapatinib PTX	11.0—5.5 8.9—4.4	diarrhea (18) neutropenia (31) leukopenia (24)	Neg
GATSBY [44]	2017	Inter	II-III	2°	345	HER2	T-DM1	HER2 pos (IHC3+, IHC2+/FISH+)	T-DM1 DTX or PTX	7.9—2.7 8.2—2.9	Anemia (26) thrombocytopenia (11)	Neg
JACOB [45]	2018	Inter	III	1°	780	HER2	Pertuzumab	HER2 pos (IHC3+, IHC2+/FISH+)	FP (XP) + trast- pertuzumab FP (XP) + trast-PBO	17.5—8.5 14.2—7.0	neutropenia (30) anemia (15) diarrhea (13)	Neg
ASLAN001-012 (NCT03130790	Ongoing	Asia	II-III	1°	400	HER1-2-3	Varlitinib	HER1-2 co-expression	mFOLFOX + varlitinib mFOLFOX + PBO	OS (PE)	NA	NA
EXPAND [46]	2013	Inter	III	1°	904	HER1	Cetuximab	All comers	XP + Cetuximab XP	9.4—4.4 10.7—5.6	Neutropenia (22) hypokalaemia (13) hypomagnesaemia (10%)	Neg
REAL3 [47]	2013	UK	III	1°	553	HER1	Panitumumab	All comers	mEOC + Panitumumab EOC	8.8—6.0 11.3—7.4	Vomiting (9) diarrhea (17) Lethargy (17)	Neg
RILOMET-1 [48]	2017	West	III	1°	609	HGF	Rilotumumab	MET pos HER2 neg	ECX + Rilotumumab ECX + PBO	8.8—5.6 10.7—6.0	Neutropenia (29) anaemia (12) fatigue (10)	Neg
METGastric [49]	2017	Inter	III	1°	562	MET	Onartuzumab	MET pos HER2 neg	mFOLFOX6 + Onartuzumab mFOLFOX6 + PBO	11.3—6.8 11.0—6.7	Neutropenia (35) hypoalbuminemia (6) pulmonary embolism (6)	Neg
FIGHT (NCT03694522)	2017	Inter	III	1°	548	FGFR2	Bemarituzumab	FGFR2 overexp/amp HER2 neg	mFOLFOX6 + Bemarituzumab mFOLFOX6 + PBO	OS (PE)	NA	NA
GRANITE-1 [50]	2013	Inter	III	2°-3°	656	mTOR	Everolimus	All comers	everolimus + BSC PBO + BSC	5.4—1.7 4.3—1.4	Anemia (16) anorexia (11) fatigue (8)	Neg
RADPAC [51]	2017	Germany	III	2°-4°	300	mTOR	Everolimus	All comers	PTX + everolimus PTX + PBO	6.1—2.2 5.0—2.0	Anemia (13) mucositis (13) diarrhea (8)	Neg
GOLD [54]	2017	Asia	III	3°	525	PARP	Olaparib	All comers	PTX + olaparib PTX + PBO	8.8 – 6.9 -	Neutropenia (30) leucopenia (10)	Neg
PARALLEL 303 (NCT03427814)	Ongoing	Inter	III	1°	540	PARP	Pamiparib	All comers	Pamiparib maintenance PBO maintenance	PFS (PE)	NA	NA
BRIGHTER [52]	2018	Inter	III	2°	714	STAT3	Napabucasin	All comers	PTX + napabucasin PTX + PBO	6.9—3.5 7.3—3.6	Diarrhea (16)	Neg
GAMMA-1 [53]	2019	West	III	1°	432	MMP9	Andecaliximab	HER2 neg	mFOLFOX6 + Andecaliximab mFOLFOX6 + PBO	12.5—7.5 11.8—7.1	Nausea (NA) diarrhea (NA) fatigue (NA) neutropenia (NA)	Neg
SPOTLIGHT (NCT03504397)	Ongoing	Inter	III	1°	550	Claudin 18.2	Zolbetuximab	CLDN18.2 pos HER2 neg	mFOLFOX6 + Zolbetuximab XELOX + PBO	PFS (PE)	NA	NA
GLOW (NCT035653507)	Ongoing	Inter	III	1°	500	Claudin 18.2	Zolbetuximab	CLDN18.2 pos HER2 neg	XELOX + Zolbetuximab mFOLFOX6 + PBO	PFS (PE)	NA	NA
AVAGAST [60]	2011	Inter	III	1°	774	VEGFA	Bevacizumab	All comers	FP (XP) + Bevacizumab FP (XP) + PBO	12.1—6.7 10.1—5.3	Neutropenia (35) anemia (10) anorexia (8)	Neg
AVATAR [59]	2015	Inter	III	1°	202	VEGFA	Bevacizumab	All comers	XP + Bevacizumab XP + PBO	11.4—6-0 10.5—6.3	Vomiting (22) neutropenia (14) hypertension (10)	Neg
REGARD [5]	2014	Inter	III	2°	355	VEGFR2	Ramucirumab	All comers	Ramucizumab + PBO PBO	5.2—2.1 3.8—1.3	Fatigue (6) hypertension (8)	Pos
RAINBOW [6]	2014	Inter	III	2°	665	VEGFR2	Ramucirumab	All comers	PTX + Ramucizumab PTX + PBO	9.6—4-4 7.4—2.9	fatigue (12) neuropathy (8) neutropenia (22) hypertension (14)	Pos
RAINFALL [58]	2019	Inter	III	1°	645	VEGFR2	Ramucirumab	HER2 neg	FP (XP) + Ramucirumab FP (XP) + PBO	11.2—5-7 10.7—5.4	Neutropenia (26) anemia (12) hypertension (10)	Neg
RAMIRIS (NCT03081143)	Ongoing	Germany	II-III	2°	429	VEGFR2	Ramucirumab	All comers	FOLFIRI + Ramucirumab PTX + Ramucirumab	OS/ORR (PE)	NA	NA
ARMANI (NCT02934464)	Ongoing	Italy	III	1°	280	VEGFR2	Ramucirumab	HER2 neg	PTX + Ramucirumab (switch maintenance) FOLFOX4, FOLFOX6, XELOX	PFS (PE)	NA	NA
RINDBeRG	Ongoing	Japan	III	3°	400	VEGFR2	Ramucirumab	All comers	IRI + Ramucirumab (beyond PD) IRI	OS (PE)	NA	NA
HENGRUI 20101208 [7]	2016	China	III	≥ 3°	267	VEGFR2	Apatinib	All comers	Apatinib PBO	6.5—2.6 4.7—1.8	HFS (8.5) hypertension (4.5)	Pos
ANGEL [40]	2019	Inter	III	≥ 3°	460	VEGFR2	Apatinib	All comers	Apatinib + BSC PBO + BSC	5. 7—2.8 5.1—1.7	Hypertension (34) HFS (26)	Neg
TJCC006 (NCT03598348)	Ongoing	China	III	1°	288	VEGFR2	Apatinib	HER2 neg	Apatinib + X maintenance after XELOX Apatinib maintenance after XELOX observation after XELOX	PFS (PE)	NA	NA
FRUTIGA (NCT02773524)	Ongoing	Inter	III	2°	544	VEGFR1/2/3	Fruquintinib	All comers	PTX + Fruquintinib PTX + PBO	OS (PE)	NA	NA
INTEGRATE II (NCT02773524)	Ongoing	Inter	III	≥ 3°	350	Multi-target	Regorafenib	All comers	Regorafenib PBO	OS (PE)	NA	NA

Ph: phase; OS: overall survival; PFS: progression-free survival; Exp: experimental arm; Cont: control arm; Res: study results according to the primary endpoint; PE: primary endpoint of the study; NA: not available; pos: positive; neg: negative; Inter: international/global; West: Western countries; HFS: hand-foot syndrome; Trast: trastuzumab; overexp: overexpression; amp: amplification; FP: 5-fluorouracil + cisplatin; XP: capecitabine-cisplatin; XELOX: capecitabine + oxaliplatin; PBO: placebo; BSC: best supportive care; PTX: paclitaxel; DTX: docetaxel; ECX: epirubicin + cisplatin + capecitabine; mEOC: modified-EOC (epirubicin + oxaliplatin + capecitabine); mFOLFOX6: modified FOLFOX6 (5-fluorouracil + leucovorin + oxaliplatin); PE: primary endpoint; CT: chemotherapy.

**Table 2:** List of ongoing phase III or II/III trials with immune checkpoint inhibitors in metastatic gastric cancer.

Study Name [reference]	Country	Ph	Line	N	Drugs (Target)	Selected Population	Study Intervention Experimental Arm/Control Arm	PE
SHR-1210-III-316 NCT04342910	China	III	2°	550	Camrelizumab (PD-1) Apatinib (VEGFR2)	All comers	Camrelizumab + apatinib paclitaxel or irinotecan	OS
ATTRACTION-04 (NCT02746796)	Asia	II-III	1°	680	Nivolumab (PD-1)	HER2 neg	CAPOX (SOX) + nivolumab CAPOX (SOX) + placebo	OS, PFS
MAHOGANY (NCT04082364)	US	II-III	1°	850	MGA012 (PD-1) MGD013 (PD-1/LAG-3) Margetuximab (HER2)	Cohort A: HER2/PD-L1 pos Cohort B: HER2 pos	Margetuximab + MGA012 XELOX (mFOLFOX6) + margetuximab + MGA012 XELOX (mFOLFOX6) + margetuximab + MGD013 XELOX (mFOLFOX6) + margetuximab XELOX (mFOLFOX6) + trastuzumab	Cohort A: ORR Cohort B: OS
KEYNOTE-063 (NCT03019588)	Asia	III	2°	360	Pembrolizumab (PD-1)	PD-L1 pos	Pembrolizumab PTX	OS, PFS
GEMSTONE-303 (NCT03802591)	China	III	1°	480	CS1001 (PD-L1)	HER2 neg	XELOX + CS1001 XELOX + placebo	OS, PFS
SHR-1210-III-311 (NCT03813784)	China	III	1°	568	SHR-1210 (PD-1) Apatinib (VEGFR2)	HER2 neg	apatinib + SHR-1210 after XELOX + SHR-1210 XELOX	OS
CIBI308E301 (NCT03745170)	China	III	1°	650	Sintilimab (PD-1)	HER2 neg	XELOX + sintilimab XELOX + placebo	OS
CheckMate 649 (NCT02872116)	Global	III	1°	2005	Pembrolizumab (PD-1) Ipilimumab (CTLA-4)	HER2 neg	Nivolumab + ipilimumab XELOX (FOLFOX) + nivolumab XELOX (FOLFOX)	OS, PFS
KEYNOTE-811 (NCT03615326)	Global	III	1°	732	Pembrolizumab (PD-1) Trastuzumab (HER2	HER2 pos	FP/XELOX/SOX + trastuzumab + pembrolizumab FP/XELOX/SOX + trastuzumab + placebo	OS, PFS
KEYNOTE-859 (NCT03675737)	Global	III	1°	780	Pembrolizumab (PD-1)	HER2 neg	FP/XELOX + pembrolizumab FP/XELOX + placebo	OS, PFS
BGB-A317-305 (NCT03777657)	Global	III	1°	720	Tislelizumab (PD-1)	HER2 neg	XELOX (FP) + tislelizumab XELOX (FP) + placebo	OS, PFS
NCT04435652	NA	II-III	2°	492	QL1604 (PD-1)	HER2 neg	QL1604 + nab-paclitaxel followed by QL1604 maintenance. paclitaxel alone	ORR, safety, OS

Ph: phase; PE: primary endpoint; XELOX: capecitabine + oxaliplatin; DCR: disease control rate; DOR: duration of response; FOLFIRI: 5-FU + leucovorin + irinotecan; FP: 5-FU + cisplatin; mFOLFOX6: modified FOLFOX6 (5-FU + leucovorin + oxaliplatin); ORR: objective response rate; OS: overall survival; PFS: progression-free survival; PTX: paclitaxel; SOX: S-1 + oxaliplatin; SP: S-1 + cisplatin; XP: capecitabine + cisplatin.

**Table 3:** List of ongoing or completed phase III or II-III trials evaluating surgery and locoregional treatments in metastatic gastric cancer.

Study name	Country	Ph	Line	N	Selected Population	Study Intervention Experimental Arm/Control Arm	PE
SURGIGAST (NCT03042169)	France	III	1°	424	GC with a single metastatic site regardless the number of lesions involving the site, in addition to the resectable PTS	CT followed by PTS resection followed by CT CT	OS
REGATTA [11]	Asian	III	1°	175	GC with resectable PTS and a single non-curable factor (2–4 liver mets of at least 1 cm, peritoneal mets in the diaphragm or peritoneum caudal to the transverse colon without massive ascites or intestinal obstruction; para-aortic lymph node metastasis above the coeliac axis or below the inferior mesenteric artery)	PTS resection followed by CT CT	OS (NEG)
EA2183 ECOG-ACRIN Cancer Research Group (NCT04248452)	US	III	any	314	GC and EGC with at most 3 metastatic lesions, in addition to the resectable PTS HER2 negative	FOLFOX alone XELOX alone FOLFOX followed by RT followed by FOLFOX XELOX followed by RT followed by XELOX	OS
FLOT5- RENAISSANCE (NCT02578368)	Germany	III	1°	271	GC with retroperitoneal lymph node mets and/or at maximum one organ involved in addition to the resectable PTS	FLOT ± Trastuzumab followed by PTS resection + metastasectomy followed by FLOT ± Trastuzumab FLOT ± Trastuzumab	OS
GASTRIPEC (NCT02158988)	Germany	III	1°	105	GC and EGC with no other than peritoneal carcinomatosis regardless previous PTS resection	Preop EOX (CXT) followed by surgery + HIPEC with cisplatin and mytomicin C followed by postop EOX (CXT) Preop EOX (CXT) followed by surgery followed by postop EOX (CXT)	OS
PERISCOPE II (NCT03348150)	Netherlands	III	any	106	GC with no other than limited peritoneal carcinomatosis (PCI < 7) and/or positive PC, in addition to PTS	Gastrectomy + cytoreductive surgery + HIPEC after SOC CT SOC CT	OS
NEO-REGATTA (NCT03001726)	China	III	any	188	GC with a single non-curable factor defined as: 2–4 hepatic mets ≤ 5 cm or positive PC or single peritoneal met with no massive ascites or PAN mets or ovary implant mets)	SLOT followed by PTS resection + metastasectomy followed by SLOT SLOT alone	OS
LP0190415 (NCT04222114)	NA	II-III	>3°	282	Peritoneal mets	Intra-peritoneal catumaxomab (EpCAM inhibitor) Investigator choice CT	OS

CT: chemotherapy; RT: radiation therapy; GC: gastric cancer; EGC: esophagogastric cancer; PTS: primary tumor site; EOX: epirubicin + oxaliplatin + capecitabine; CXT: cisplatin + capecitabine + trastuzumab; met: metastasis; PC: peritoneal citology; PCI: peritoneal cancer index; NEG: negative; PAN: para-aortic lymph node; SLOT: S1 + oxaliplatin + docetaxel; SOC: standard of care; EpCAM: epithelial cell adhesion molecule.
